# Genetic Evidence Reveals Causal Effect of Circulating Proteome on Random Glucose: A Mendelian Randomization Study

**DOI:** 10.1155/jdr/6662650

**Published:** 2026-03-03

**Authors:** Ziyuan Shen, Xing Xing, Xiaoyue Zhang, Jianan Zhu, Yining Wang, Guoqi Cai

**Affiliations:** ^1^ Department of Epidemiology and Biostatistics, School of Public Health, Anhui Medical University, Hefei, Anhui, China, ahmu.edu.cn; ^2^ Department of Biostatistics, Johns Hopkins Bloomberg School of Public Health, Baltimore, Maryland, USA, jhu.edu; ^3^ Department of Biostatistics, New York University School of Global Public Health, New York, New York, USA; ^4^ Menzies Institute for Medical Research, University of Tasmania, Hobart, Tasmania, Australia, utas.edu.au

**Keywords:** circulating proteome, diabetes, Mendelian randomization, random glucose

## Abstract

**Background:**

Random glucose (RG) testing provides greater flexibility and convenience, enabling real‐time evaluation of blood glucose levels without the need to consider recent dietary intake. This study was aimed at identifying drug targets using the evidence from circulating proteins associated with RG from genome‐wide association studies (GWASs)

**Methods:**

Using two‐sample Mendelian randomization (MR) with circulating protein data from nine GWAS, we revealed potential causal relationships between these proteins and RG. A framework of sensitivity analyses was performed to assess the robustness and credibility of the evidence.

**Results:**

In the *cis*‐protein quantitative trait loci (pQTLs) and the combined *cis*/*trans*‐pQTLs analyses, 12 and 31 proteins demonstrated causal effects on RG, respectively. Enrichment analysis revealed that proteins prioritized by *cis*‐MR were enriched in the carbohydrate catabolic process, collagen‐containing extracellular matrix, and peptidase regulator activity. For all MR‐prioritized proteins, pathways were enriched in those related to the maintenance of location, secretory granule lumen, sulfuric ester hydrolase activity, and regulation of lipolysis in adipocytes. Notably, approximately half of these proteins (including PCSK1, PPY, and VWF) were recognized as druggable or existing drug targets.

**Conclusions:**

This study identified proteins causally linked to RG, emphasizing their potential role in the development of therapeutic interventions for metabolic disorders, particularly those involving glucose regulation.

## 1. Introduction

Glucose homeostasis is essential for maintaining metabolic equilibrium and ensuring the proper functioning of various bodily systems. This regulatory process involves complex interactions among hormones and biochemical pathways that maintain stable blood glucose levels. Impaired regulation of glucose homeostasis plays a pivotal role in the development of major metabolic conditions, including obesity and Type 2 diabetes (T2D) [1]. While fasting blood glucose (FBG) and postprandial blood glucose (PPBG) measurements are traditional methods for monitoring blood glucose levels, FBG requires prior fasting, and both are significantly influenced by the diet consumed [2]. In contrast, random glucose (RG) provides greater flexibility and convenience, enabling real‐time evaluation of blood glucose levels without the need to consider recent dietary intake [3]. Moreover, RG reflects the integrated physiological regulation of glucose across various organ systems, making it an important index for revealing the pathophysiology of metabolic disorders.

The plasma proteome reflects not only normal physiological processes but also pathogenic biological activities [4], making human blood plasma an invaluable resource for exploring an individual′s proteome in various states of health and disease [5]. The circulating proteome represents a dynamic collection, maintained by the continuous inflow and outflow of proteins produced by various tissues and cells throughout the body [6]. Genome‐wide association studies (GWASs) focusing on plasma protein concentrations have identified variations attributable to common single‐nucleotide polymorphisms (SNPs), thereby establishing protein quantitative trait loci (pQTLs) [7–10]. Using Mendelian randomization (MR) to analyze these pQTLs offers a robust approach for deducing causal links between alterations in protein levels and disease, particularly when a pQTL is located near its corresponding gene (*cis*) [11, 12].

Recent studies have highlighted the important role of circulating proteins in glucose regulation. Inflammatory circulating proteins, such as IL‐6, can disrupt insulin signaling, leading to insulin resistance [13, 14]. Insulin resistance is a key component of the pathogenesis of T2D, characterized by impaired insulin signal transmission and ineffective blood glucose control [15]. Conversely, adiponectin, a protein secreted by adipose tissue, has demonstrated a potential to enhance insulin sensitivity and help prevent the development of diabetes [16]. However, the causal impact of circulating proteins on RG remains unclear. Clarifying the associations through which circulating proteins influence glucose homeostasis is crucial for understanding metabolic health. Therefore, this study was aimed at identifying circulating proteins that were causally related to RG levels through MR analysis.

## 2. Materials and Methods

### 2.1. Study Design

The causal relationship between circulating proteins and RG was assessed using a two‐sample MR analysis. MR employs genetic variation as instrumental variables (IVs) to represent risk factors. These IVs must satisfy three critical assumptions [17]: (1) it is strongly associated with the exposure, (2) it is independent of any confounders linking exposure and outcome, and (3) it influences the outcome exclusively through the exposure, without alternative pathways. This genetic association study was conducted according to the Strengthening the Reporting of Observational Studies in Epidemiology Using Mendelian Randomization (STROBE‐MR) [18]. As this MR analysis used publicly available data from previously published GWASs that had already received ethical approval and participant consent, separate ethical approval for this study was waived by the Ethics Review Board of Anhui Medical University.

### 2.2. GWAS Data Sources for Circulating Proteins and Selection of IVs

Data on circulating proteins were obtained from nine proteomic GWAS, with screening criteria including a sample size greater than 500 and at least 50 measured proteins (Supporting Information 2: Table S1) [7, 8, 19–25]. Serum pQTLs were used as candidate IVs according to the following steps: (1) SNPs were selected based on their association with any protein, adhering to the *p* value thresholds recommended in the respective studies (Supporting Information 2: Table S1); (2) SNPs located within the major histocompatibility complex (MHC) region of the human genome (Chromosome 6, spanning from 26 to 34 Mb) were excluded due to the complex patterns of linkage disequilibrium (LD) in this region [26]; (3) LD clumping was conducted with a threshold of *r* ^2^ > 0.01 and a maximum distance of 500 kb both upstream and downstream to identify independent pQTLs for each protein; and (4) instruments associated with five or more proteins were excluded due to their high degree of pleiotropy.

In this study, instruments were categorized into *cis*‐pQTLs and *trans*‐pQTLs. *cis*‐pQTLs were defined as pQTLs located within a 500‐kb window surrounding the corresponding protein‐coding gene sequences. A pQTL was considered *trans*‐acting if it was located more than 500 kb from the gene encoding the corresponding protein.

### 2.3. Data Sources for RG

The RG GWAS summary statistics were obtained from the Meta‐Analyses of Glucose and Insulin‐related traits Consortium (MAGIC) [27], which included up to 476,326 individuals from 70 cohorts. Our analysis focused on approximately 458,862 individuals who did not have a diagnosis of diabetes and were not receiving glucose‐lowering medications. All participants were of European ancestry. Although individual‐level demographic data were not publicly available, detailed cohort‐level characteristics such as age distribution, sex ratio, and phenotype definitions can be found in Supporting Information 2: Table S1 of the original GWAS publication. In general, participants were drawn from population‐based cohorts in Europe or other Western countries. There is minor sample overlap between the protein and RG GWAS datasets, primarily due to the inclusion of the deCODE cohort in both. The deCODE samples account for approximately 3.9% of the total RG GWAS population. Although we could not confirm individual‐level overlap, the overall proportion of shared samples is likely small.

### 2.4. MR and Sensitivity Analyses

In this study, *cis*‐pQTLs and all pQTLs were, respectively, used as the instruments for constructing the MR analysis. Inverse variance weighting (IVW) [28] and Wald ratio analyses were employed to evaluate the causal relationships where either a single pQTL or multiple pQTLs were present [29]. Sensitivity analysis was conducted to mitigate the influence of pleiotropy, excluding instruments associated with multiple proteins. Additionally, further sensitivity analyses focused on addressing potential issues of heterogeneity and horizontal pleiotropy associated with the instruments. When heterogeneity among multiple instruments was identified using Cochrane′s *Q*‐tests, the weighted median approach was applied. This method is robust to up to 50% invalid instruments [30]. Furthermore, the MR–Egger method was used to adjust for horizontal pleiotropy if detected [30, 31]. For each analyzed outcome, Bonferroni correction was applied to adjust significance thresholds, setting the *p* values at 0.05 divided by the number of proteins tested (Supporting Information 2: Table S2).

### 2.5. Colocalization Analysis

To assess the possibility of confounding due to LD, we investigated the associations between *cis*‐pQTLs and all pQTLs of MR‐prioritized proteins with glycemic traits and determined whether these pQTLs were in LD with different causal variants for these traits. For this purpose, Bayesian colocalization analysis was performed using the “coloc” package in R software (http://www.Rproject.org). This analysis was aimed at determining the posterior probability that a single variant within a genomic locus simultaneously affects both protein levels and glycemic traits [32]. The analysis yields posterior probabilities for five different hypotheses (H0, H1, H2, H3, and H4). We focused on SNPs located within a 1‐Mb range of the *cis*‐pQTLs and all pQTLs. Strong evidence of colocalization was defined as a posterior probability for H4 greater than 0.8 [12]. However, Bayesian colocalization analysis operates under the assumption that there is only one shared causal SNP within a genetic locus, although in reality, a locus may contain multiple causal SNPs [33].

### 2.6. Steiger Filtering Analysis

The Steiger filtering approach was used to examine the MR associations that passed the multiple‐testing threshold to determine if reverse causality could skew the results (‘TwoSampleMR’ package in R software) [34]. The outcomes are categorized as follows: labeled as “true” when the effect direction is from exposure to outcome with a *p* value less than 0.05, marked “false” if the effect is reversed with a *p* value less than 0.05, and designed “uncertain” if the *p* value is 0.05 or higher.

### 2.7. Assessment of Protein‐Altering Variants (PAVs) for *cis*‐pQTLs

In affinity‐based proteomic analyses, the recognition of stable binding epitopes is a critical prerequisite for accurate protein detection [24]. Genetic variants, referred to as PAVs, that alter protein structures could lead to the creation of apparent *cis*‐pQTLs due to changes in aptamer binding, which may not reflect true quantitative changes in protein levels. To evaluate the potential for aptamer binding effects, *cis*‐pQTLs showing evidence in MR analyses were examined to determine if they were PAVs or in LD with PAVs (with *r* ^2^ ≥ 0.8). Variants were classified as protein‐altering if they were annotated with any of the following consequences: coding sequence changes, frameshift alterations, in‐frame deletions or insertions, missense mutations, splice site disruptions (acceptor, donor, or region), or changes affecting start and stop codons (lost or gained) [7].

### 2.8. Expression Quantitative Trait Locus (eQTL) Assessment

To explore the potential mechanisms by which pQTLs influence plasma protein levels, their overlap with eQTLs was examined through direct genetic lookups. For pQTLs supported by MR evidence, both the variant in question and its proxies (*r* ^2^ ≥ 0.8) were evaluated to determine if they had significant corresponding eQTLs with the same allelic direction. This analysis used data from the Genotype‐Tissue Expression (GTEx) Portal (https://www.gtexportal.org) [35].

### 2.9. Protein–Protein Interaction (PPI) and Functional Enrichment Analysis

PPI networks were created for individual proteins, providing function prediction hypotheses. PPI analysis was conducted using the STRING database (https://string-db.org). Gene Ontology (GO) and Kyoto Encyclopedia of Genes and Genomes (KEGG) pathway analyses were conducted to elucidate the biological function of the proteins (“ClusterProfiler” package in R software).

### 2.10. Mapping of the MR‐Prioritized Proteins With Drug Targets

The human plasma proteome is recognized as a crucial source of therapeutic targets [7]. In this study, the MR‐prioritized proteins (with evidence from two‐sample MR) were aligned with 4479 genes, identified by Finan et al. as the drugged or druggable genome [36]. These genes were categorized into three distinct tiers based on their status in the drug development process. A total of 1427 genes were classified into Tier 1, encompassing efficacy targets of both approved small‐molecule drugs and biotherapeutics, as well as candidates currently in clinical development. Tier 2 encompasses targets known to bind with biologically active small molecules and drug‐like partners, along with targets sharing at least 50% identity (across more than 75% of their sequence) with approved drug targets. Tier 3, consisting of 2370 genes, includes secreted or extracellular proteins, proteins that are somewhat similar to approved drug targets, and key drug‐related genes not classified in Tiers 1 or 2. This third tier is further subdivided into Tier 3A, which contains genes located within ± 50 kbp of a GWAS SNP and have an extracellular location, and Tier 3B, which includes the remaining genes in Tier 3. In our approach to further investigate drug targets, we employed a biotherapeutic (monoclonal antibody/enzyme or other protein) to gain deeper insights and confirm the robustness of our findings. We evaluated the priority tier level for each druggable gene, the types of protein products targeted, and whether its protein product is currently known or anticipated to be a drug target.

## 3. Results

### 3.1. Overview of Genetic Instrument Selection

**Figure 1:**
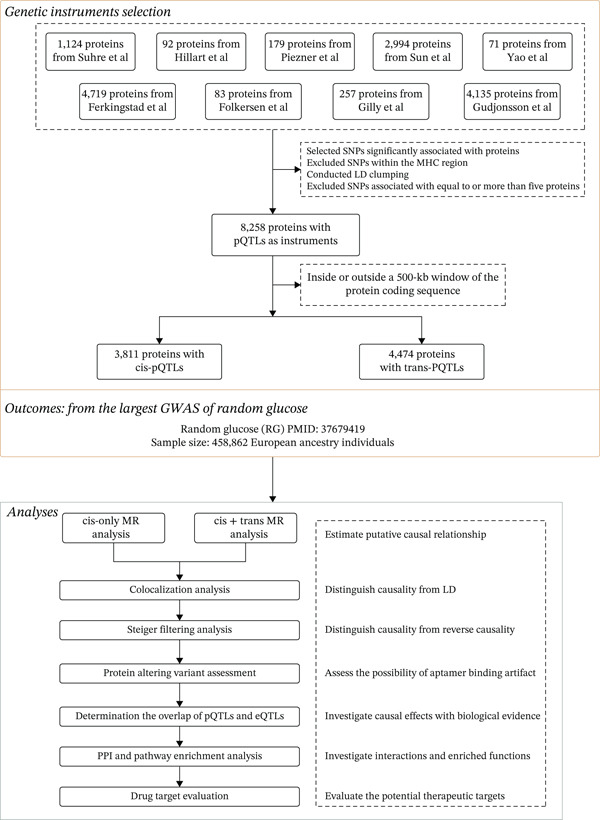
Flowchart of Mendelian randomization–based study for the causal effect of circulating proteome on random glucose. SNPs, single‐nucleotide polymorphisms; pQTL, protein quantitative trait loci; LD, linkage disequilibrium; PPI, protein–protein interaction.

Following the selection process depicted in Figure 1, a total of 8258 pQTLs (4421 proteins) were identified from nine GWAS (Supporting Information 2: Table S3). The plasma protein apparatus comprised 3811 *cis*‐pQTLs (1558 unique proteins) and 4474 *trans*‐pQTLs (1763 unique proteins).

### 3.2. The Causal Effects of Plasma Proteins on RG

Initially, we employed *cis*‐pQTLs as instruments for MR analysis to investigate the causal effects of plasma proteins on RG (Supporting Information 2: Table S4). Following Bonferroni correction, 14 unique proteins exhibited significant causal associations with RG: causal associations with RG were identified for 14 unique proteins (Table 1 and Figure 2a). Among them, YWHAB, HDGF, CREB3L4, and BPNT1 were positively associated with RG levels. In contrast, PGM1 and SPINK4 were negatively associated with RG. Subsequent MR analyses were conducted using all pQTLs (Supporting Information 2: Table S6), revealing that 31 unique proteins were associated with RG after correction (Figure 2b). Sensitivity analyses reinforced the reliability of these findings (Supporting Information 2: Tables S5 and S7).

**Table 1:** Protein–phenotype associations identified by Mendelian randomization using cis‐only pQTLs.

Uniport	Protein	Direction
A6NHS7	MANSC domain containing 4 (MANSC4)^a,b,c^	Negative
P29120	Proprotein convertase subtilisin/kexin type 1 (PCSK1)^a,b,c^	Positive
P31946	Tyrosine 3‐monooxygenase (YWHAB)^a^	Positive
P51858	Heparin binding growth factor (HDGF)^a^	Positive
Q13137	Calcium binding and coiled‐coil domain 2 (CALCOCO2) ^a^	Negative
Q15113	Procollagen C‐endopeptidase enhancer (PCOLCE)^a,b,c^	Positive
Q8TEY5	CAMP responsive element binding protein 3 like 4 (CREB3L4)^a,b,c^	Positive
Q9NXS2	Glutaminyl‐peptide cyclotransferase like (QPCTL)^a,b^	Negative
O60575	Serine peptidase inhibitor Kazal type 4 (SPINK4)^c^	Negative
O95861	3 ^′^(2 ^′^), 5 ^′^‐Bisphosphate nucleotidase 1 (BPNT1)^c^	Positive
P31946	Tyrosine 3‐monooxygenase (YWHAB)^c^	Positive
P36871	Phosphoglucomutase 1 (PGM1)^c^	Negative
P51858	Heparin binding growth factor (HDGF)^c^	Positive
Q9UKK9	Nudix hydrolase 5 (NUDT5)^c^	Positive

^a^Alexander Gudjonsson et al.

^b^Benjamin B. Sun et al.

^c^Egil Ferkingstad et al.

Figure 2Volcano plot. (a) cis‐only Mendelian randomization analysis. (b) cis/trans‐Mendelian randomization analysis.

**Figure:**
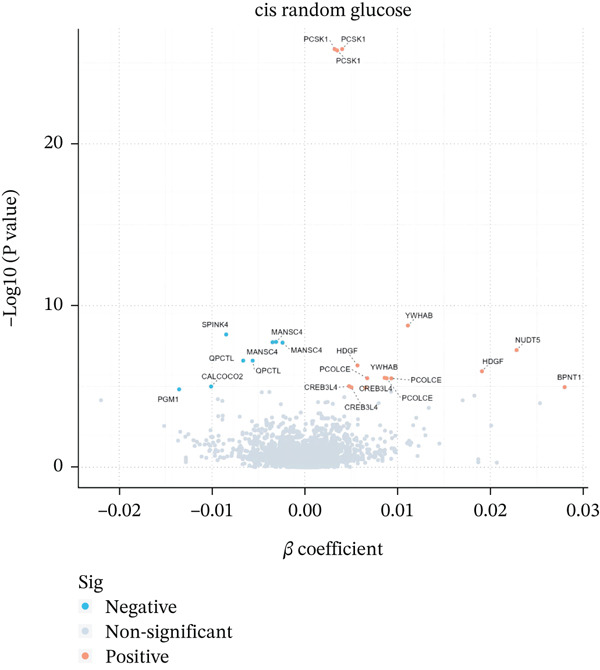
(a)

**Figure:**
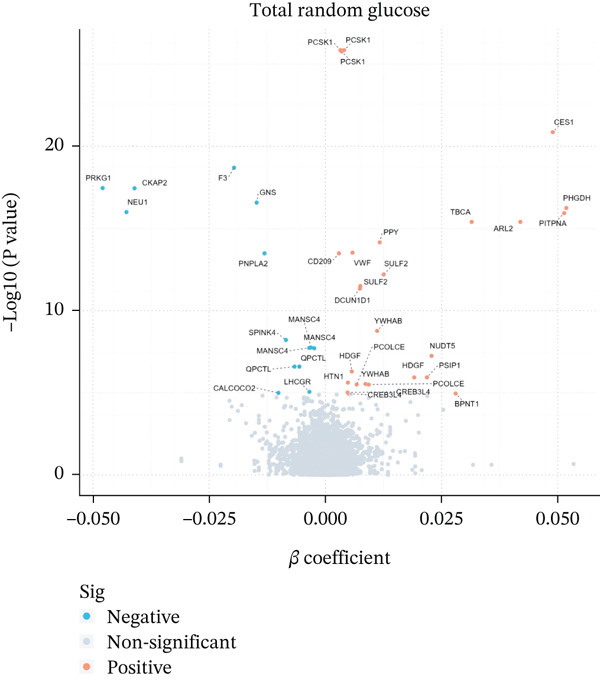
(b)

### 3.3. Colocalization of the pQTLs With RG

The colocalization analysis of pQTLs identified via *cis*‐only MR analyses within RG loci revealed that eight protein associations showed strong evidence of colocalization (PPH4 ≥ 0.8; Supporting Information 2: Table S8), including A6NHS7, Q13137, Q8TEY5, Q9NXS2, O95861, P31946, P36871, and P51858.

For all pQTLs identified by MR analysis, eight (i.e., A6NHS7, Q13137, Q8TEY5, Q9NXS2, O95861, P31946, Q99519, and P51858) associations that underwent colocalization analysis exhibited strong evidence of colocalization (Supporting Information 2: Table S9).

### 3.4. Results of Steiger Filtering Analysis

Steiger filtering analysis was conducted to evaluate the directionality of the associations identified through MR. For the unique proteins prioritized in the *cis*‐only MR analysis, all exhibited consistent causal directions from protein to RG, with Steiger *p* values ranging from 0 to 1.7 × 10^−112^ (Supporting Information 2: Table S10).

Similarly, among the 31 proteins identified using all pQTLs, Steiger analysis confirmed directional consistency for all associations (Supporting Information 2: Table S11). No associations were flagged as uncertain or reversed in either analyses.

### 3.5. Assessment of PAV

Among the *cis*‐pQTLs of MR‐prioritized proteins, 24 variants were evaluated for potential protein‐altering effects. A total of five proteins were found to possess or be in high LD (*r* ^2^ > 0.8) with PAVs, including PCSK1, HDGF, CALCOCO2, QPCTL, and PGM1 (Supporting Information 2: Table S12).

### 3.6. Investigating the Overlap of pQTLs and eQTLs

In the MR analyses restricted to *cis*‐pQTLs, 11 variants also acted as significant eQTLs in at least one tissue and showed consistent directions of effect (Supporting Information 2: Table S13). No overlap was found in the MR analyses with all pQTLs.

### 3.7. Enrichment Analysis of the MR‐Prioritized Proteins

The PPI network identified potential interactions among proteins analyzed through MR analysis. Notably, PCSK1 and MANSC4 demonstrated a direct interaction using *cis*‐pQTLs (Supporting Information 1: Figure S1). VWF emerged as a central node within the MR‐prioritized proteins, interacting with multiple proteins, including F3 and PRKG1 (Supporting Information 1: Figure S2). GO pathway enrichment analysis showed that proteins prioritized by *cis*‐MR were mainly enriched in the carbohydrate catabolic process, collagen‐containing extracellular matrix, and peptidase regulator activity (Figure 3a, Supporting Information 2: Table S14). KEGG analysis revealed the *cis*‐MR prioritized proteins were mainly enriched in purine metabolism, hepatitis B, and viral carcinogenesis (Figure 3c, Supporting Information 2: Table 16). Pathway enrichment analysis for all MR‐prioritized proteins revealed predominant processes like the maintenance of location, secretory granule lumen, sulfuric ester hydrolase activity, and regulation of lipolysis in adipocytes (Figure 3b,d, Supporting Information 2: Tables S15 and S17).

Figure 3Enriched pathways of the proteins prioritized by (a, b) cis‐only and (c, d) cis/trans Mendelian randomization.

**Figure:**
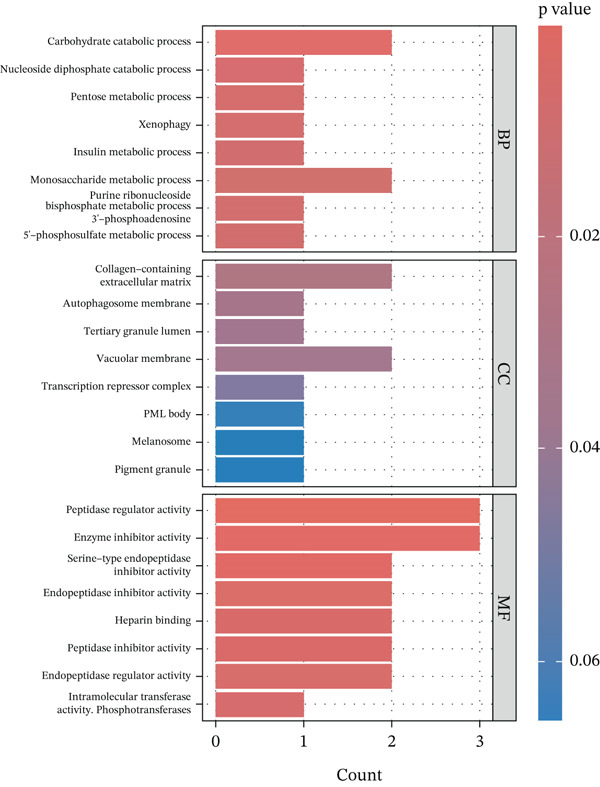
(a)

**Figure:**
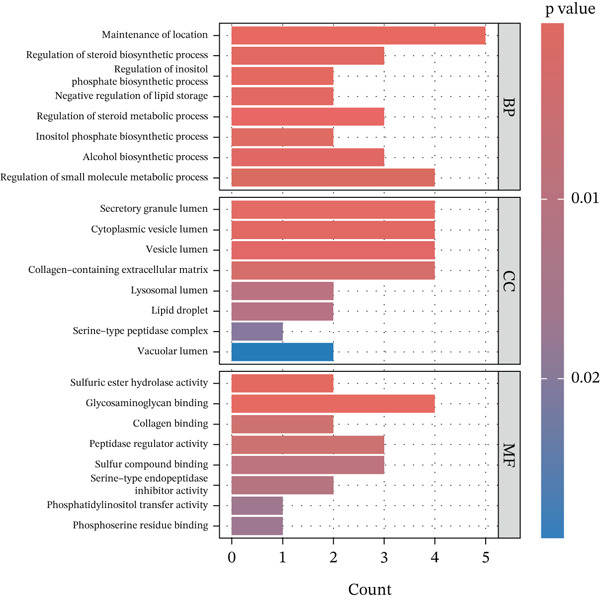
(b)

**Figure:**
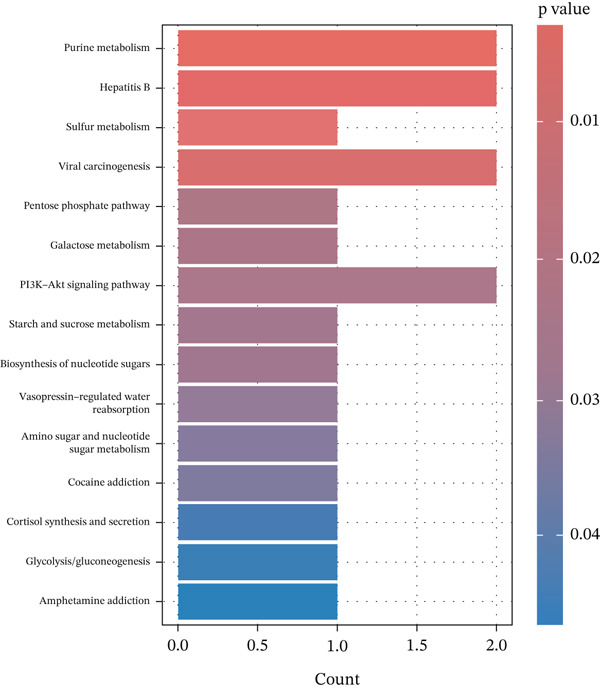
(c)

**Figure:**
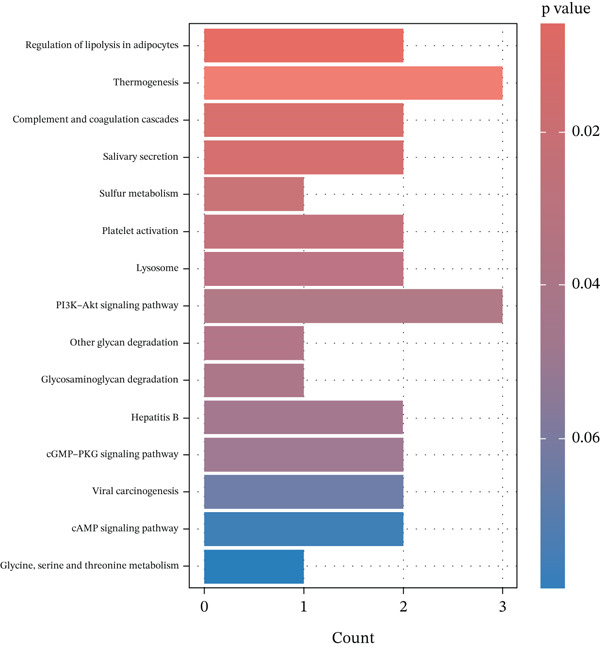
(d)

### 3.8. Druggability Evaluation on the MR‐Prioritized Proteins

According to the classification scheme by Finan et al., four proteins were categorized as Tier 1 (i.e., Von Willebrand factor, coagulation factor III, luteinizing hormone/choriogonadotropin receptor, and carboxylesterase 1; Table 2). Additionally, one protein was classified under Tier 2 (i.e., protein kinase CGMP‐dependent 1). At the Tier 3A and Tier 3B levels, six and four proteins were identified, respectively. Using the Therapeutic Target Database, we identified 14 MR‐prioritized proteins as existing or potential drug targets (i.e., PCSK1, HDGF, CD209, PHGDH, ARL2, PRKG1, VWF, CES1, LHCGR, NEU1, F3, SULF2, PPY, and PSIP1). Six were literature‐reported targets (PCSK1, HDGF, CD209, PHGDH, ARL2, PRKG1), five were successful targets with approved or clinical‐use drugs (VWF, CES1, LHCGR, NEU1, and F3), two were under clinical trials (SULF2 and PPY), and one was a patented‐recorded target (PSIP1, Supporting Information 2: Table S18).

**Table 2:** List of the MR‐prioritised proteins that were drug targets or druggable.

Uniprot	Protein	Druggable genes from Finan et al.	Therapeutic target database
		Druggability_tier	Target type
P29120	Proprotein convertase subtilisin/kexin type 1 (PCSK1)	Tier 3A	Literature‐reported target
P51858	Heparin binding growth factor (HDGF)	Tier 3A	Literature‐reported target
O60575	Serine peptidase inhibitor kazal type 4 (SPINK4)	Tier 3A	/
O95861	3 ^′^(2 ^′^), 5 ^′^‐Bisphosphate nucleotidase 1 (BPNT1)	Tier 3B	/
P04275	Von Willebrand factor (VWF)	Tier 1	Successful target
P15515	Histatin 1 (HTN1)	Tier 3B	/
P15586	Glucosamine (N‐acetyl)‐6‐sulfatase (GNS)	Tier 3B	/
Q8IWU5	Sulfatase 2 (SULF2)	/	Clinical trial target
P01298	Pancreatic polypeptide (PPY)	Tier 3A	Clinical trial target
Q9NNX6	CD209 molecule (CD209)	Tier 3B	Literature‐reported target
O43175	Phosphoglycerate dehydrogenase (PHGDH)	/	Literature‐reported target
O75475	PC4 and SRSF1 interacting protein 1 (PSIP1)	/	Patented‐recorded target
P20742	PZP alpha‐2‐macroglobulin like (PZP)	Tier 3A	/
P22888	Luteinizing hormone/choriogonadotropin receptor (LHCGR)	Tier 1	Successful target
P23141	Carboxylesterase 1 (CES1)	Tier 1	Successful target
P36404	ADP ribosylation factor like GTPase 2 (ARL2)	/	Literature‐reported target
Q13976	Protein kinase CGMP‐dependent 1 (PRKG1)	Tier 2	Literature‐reported target
Q99519	Neuraminidase 1 (NEU1)	Tier 3A	Successful target
P13726	Coagulation factor III, tissue factor (F3)	Tier 1	Successful target

## 4. Discussion

In this study, we used a comprehensive and integrative approach to identify causal associations between plasma proteins and RG across nine proteomics GWAS datasets. Our two‐sample MR analyses revealed that 14 unique proteins were causally associated with RG using *cis*‐pQTLs, and 31 unique proteins were identified via all pQTLs. Furthermore, we elucidated potential regulatory pathways and therapeutic targets associated with these proteins. These findings suggest that the causal links identified between plasma proteins and RG could facilitate the development of novel biomarkers and personalized treatment strategies for metabolic disorders, including diabetes.

The current MR analysis employing *cis*‐pQTL identified significant causal relationships between the upregulation of tyrosine 3‐monooxygenase (YWHAB) and three other proteins (HDGF, CREB3L4, and BPNT1) with RG. These findings were further substantiated by robust results from both colocalization analysis and Steiger filtering analysis. The upregulation of YWHAB aligns with findings regarding the metabolic influence of the 14‐3‐3 protein family [37]. Notably, in the Bama longevity area, higher levels of these proteins, including YWHAB, have been observed in offspring exhibiting normal functional urea nitrogen levels. Furthermore, the proteins HDGF, CREB3L4, and BPNT1 also demonstrated significant causal links with RG levels, involved in various pathways contributing to glucose homeostasis and metabolic health [38, 39]. These proteins highlight a complex network of metabolic regulation, suggesting that they could serve as potential biomarkers for understanding and managing glucose homeostasis in metabolic disorders. The downregulation of four proteins suggested a disruption in normal metabolic processes, potentially influencing diabetes development and progression. Phosphoglucomutase 1 (PGM1) plays a central role in glycogen metabolism by reversibly modulating both its synthesis and breakdown under physiological conditions [40]. Glutaminyl‐peptide cyclotransferase‐like (QPCTL) is associated with the immune response and inflammation, both critical factors in metabolic health. These proteins indirectly influence metabolic processes that are crucial for insulin resistance and T2D. Bresser et al. [41] demonstrated that QPCTL regulates the abundance of macrophages and monocytes in the tumor microenvironment. This regulation impacts inflammatory signatures that are known to affect metabolic health, further connecting immune response mechanisms with metabolic dysfunction.

In this study, MR analysis using all pQTLs also identified causal links between several plasma proteins with RG. To enhance the accuracy of causal inference, we further conducted colocalization analysis and Steiger filtering analysis, similar to recent methodological frameworks adopted in large‐scale proteome‐wide MR studies [10]. Through colocalization analysis and Steiger filtering analysis, we found that eight proteins have associations with RG that remain robust under multiple statistical validations. Among these, the functions of seven proteins have already been discussed, highlighting their roles in metabolism. Neuraminidase 1 (NEU1) is known for its role in the lysosomal catabolism of sialylated glycoconjugates in cells, but emerging evidence suggests that it could play a key role in metabolic disorders [42]. Previous research has demonstrated that the insulin receptor is one of the receptors modulated by NEU1, suggesting that NEU1 could play a crucial role in regulating insulin sensitivity and glucose homeostasis [43]. This modulation of insulin receptor activity by NEU1 indicates its potential as a novel target for therapeutic interventions aimed at improving insulin sensitivity and preventing the progression of metabolic diseases.

In our study, MR analysis successfully identified several plasma proteins as therapeutic targets. Among the identified targets, pancreatic polypeptide (PPY) was identified for obesity that could impact obesity treatments. Both PPY and PYY are part of the neuropeptide Y (NPY) family, known for their roles in regulating appetite and food intake [44]. PYY is secreted by intestinal L cells as PYY1‐36 and then transformed into PYY3‐36 by DPP4, binding to the Y2 receptor to decrease food intake [45]. Similarly, PPY, produced in the pancreas, primarily acts on the Y4 receptor [46]. This action of PPY helps to further regulate appetite by slowing gastric emptying and enhancing satiety signals, complementing the effects of PYY in maintaining energy balance. These results highlight the potential of targeting PPY to develop novel treatments aimed at reducing obesity by controlling appetite and improving energy homeostasis.

This study has several strengths. First, we identified several novel causal associations between plasma proteins and RG, contributing to the understanding of diabetes mechanisms and offering new perspectives for prevention and treatment. Second, by employing two‐sample MR analysis, this study minimizes confounding biases, thereby enhancing the causal inference between circulating proteins and RG. In addition, previous methodological work on MR has demonstrated that substantial bias due to sample overlap mainly arises when the proportion of shared samples is large and the instruments are weak [47], indicating that the risk of overlap‐induced bias in our study is minimal. Limitations of the current study are worth noting. First, the summary‐level data from the previous GWAS included only European ancestries, potentially limiting generalizability to other ancestries. Second, our analysis focused on circulating proteins, which encompass both intentionally secreted and incidentally leaked proteins. However, the concentrations of these proteins in the bloodstream may not reflect their levels in specific cells and tissues.

## 5. Conclusions

In conclusion, our results provide evidence that specific circulating proteins are causally associated with RG. Future research is necessary to clarify the pathophysiological roles of these proteins and to elucidate the molecular mechanisms underlying their effects on RG.

**Nomenclature**
eQTL: expression quantitative trait loci
FBG: fasting blood glucose
GO: Gene Ontology
GWASs: genome‐wide association studies
KEGG: Kyoto Encyclopedia of Genes and Genomes
IVs: instrumental variables
LD: linkage disequilibrium
MHC: major histocompatibility complex
MR: Mendelian randomization
PAVs: protein‐altering variants
PPBG: postprandial blood glucose
PPI: protein–protein interaction
pQTL: protein quantitative trait loci
RGT: random glucose testing
SNPs: single‐nucleotide polymorphisms
T2D: Type 2 diabetes

## Author Contributions

**Ziyuan Shen:** writing—original draft and formal analysis. **Xing Xing:** writing—original draft and formal analysis. **Xiaoyue Zhang:** data curation. **Jianan Zhu:** data curation. Yining Wang: data curation. **Guoqi Cai:** conceptualization, supervision, an writing— review and editing. **Ziyuan Shen, Xing Xing,** and **Xiaoyue Zhang** are joint first authors.

## Funding

This work was supported by the National Natural Science Foundation of China, 10.13039/501100001809, 82103933, and Scientific Research Level Upgrading Project of Anhui Medical University, 2021xkjT006. Open access publishing facilitated by University of Tasmania, as part of the Wiley ‐ University of Tasmania agreement via the Council of Australasian University Librarians.

## Ethics Statement

The ethical approval and consent information for these data can be found in the original publication.

## Consent

The authors have nothing to report.

## Conflicts of Interest

The authors declare no conflicts of interest.

## Supporting Information

Additional supporting information can be found online in the Supporting Information section.

## Supporting information


**Supporting Information 1** Figure S1: Protein–protein interaction networks of the *cis*‐only Mendelian randomization‐prioritized proteins. Figure S2: Protein–protein interaction networks of the *cis*/*trans*‐Mendelian randomization‐prioritized proteins.

**Supporting Information 2** Table S1: Source information of the instrumental variables. Table S2: Bonferroni *p* value threshold for each outcome. Table S3: Information of the instrumental variables. Table S4: Main analysis results of the *cis*‐only MR. Table S5: Sensitivity analyses results of *cis*‐only MR. Table S6: Main analysis results of the *cis* + *trans*‐MR. Table S7: Sensitivity analyses results of *cis* + *trans*‐MR. Table S8: Colocalization of the pQTLs that prioritized by the *cis*‐only MR analyses with RG loci. Table S9: Colocalization of the pQTLs that prioritized by the main analyses of *cis* + *trans*‐MR with RG loci. Table S10: Steiger filtering analysis for associations identified by *cis*‐only MR. Table S11: Steiger filtering analysis for associations identified by main analyses of *cis* + *trans*‐MR. Table S12: PAV assessment for the *cis*‐pQTLs with MR evidence. Table S13: Overlaping of pQTLs that prioritized by *cis*‐only MR with eQTLs. Table S14: GO pathway enrichment analysis for proteins prioritized by *cis*‐only MR. Table S15: GO pathway enrichment analysis for proteins prioritized by *cis* + *trans*‐MR. Table S16: KEGG pathway enrichment analysis for proteins prioritized by *cis*‐only MR. Table S17: KEGG pathway enrichment analysis for proteins prioritized by *cis* + *trans*‐MR. Table S18: Drug targets of the MR‐prioritized proteins.

## Data Availability

The data that support the findings of this study are available from the corresponding author upon reasonable request.
