# Supplementary material for: Genetic Evidence Reveals Causal Effect of Circulating Proteome on Random Glucose: A Mendelian Randomization Study
Source: J Diabetes Res. 2026 Mar 3;2026:6662650. doi: 10.1155/jdr/6662650 (PMC12957537; doi:10.1155/jdr/6662650)
Supplement: Supplementary file 1 — Supporting Information 1 Figure S1: Protein–protein interaction networks of the cis‐only Mendelian randomization‐prioritized proteins. Figure S2: Protein–protein interaction networks of the cis/trans‐Mendelian randomization‐prioritized proteins. [file JDR-2026-6662650-s001.docx]

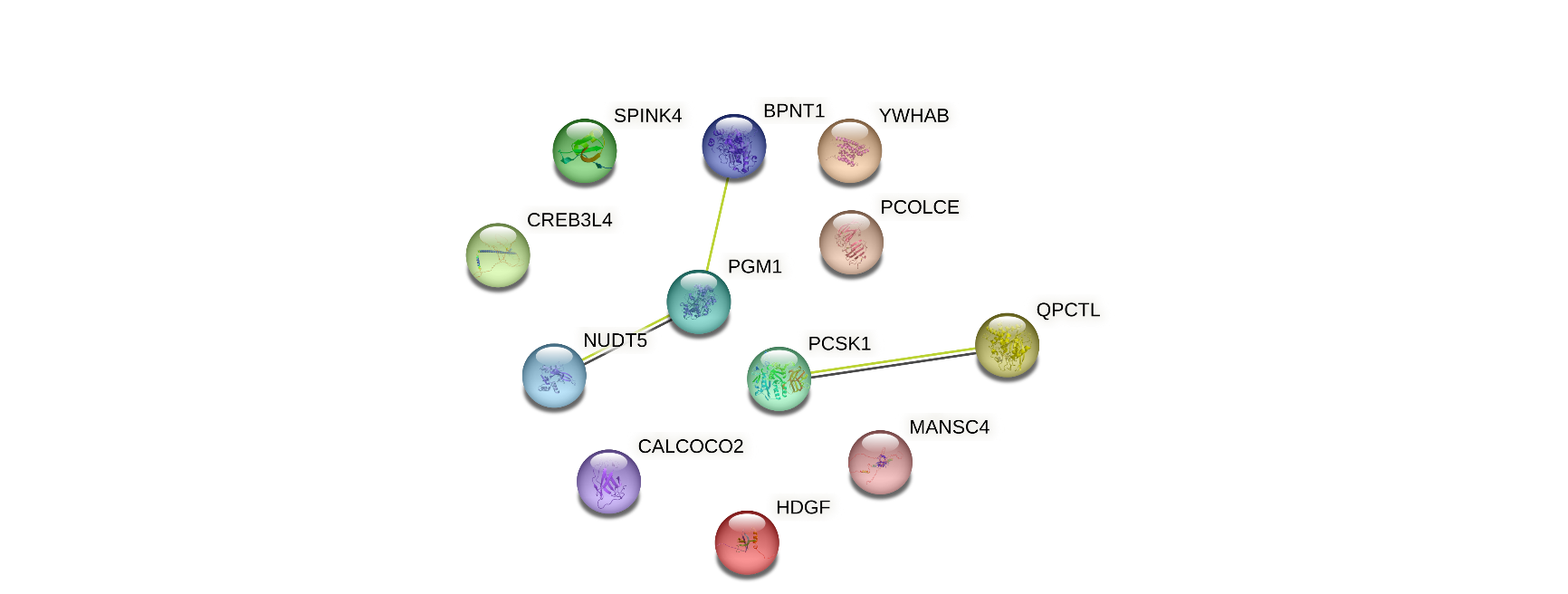


Supplementary Figure 1: Protein-protein interaction networks of the cis-only Mendelian randomization-prioritized proteins.


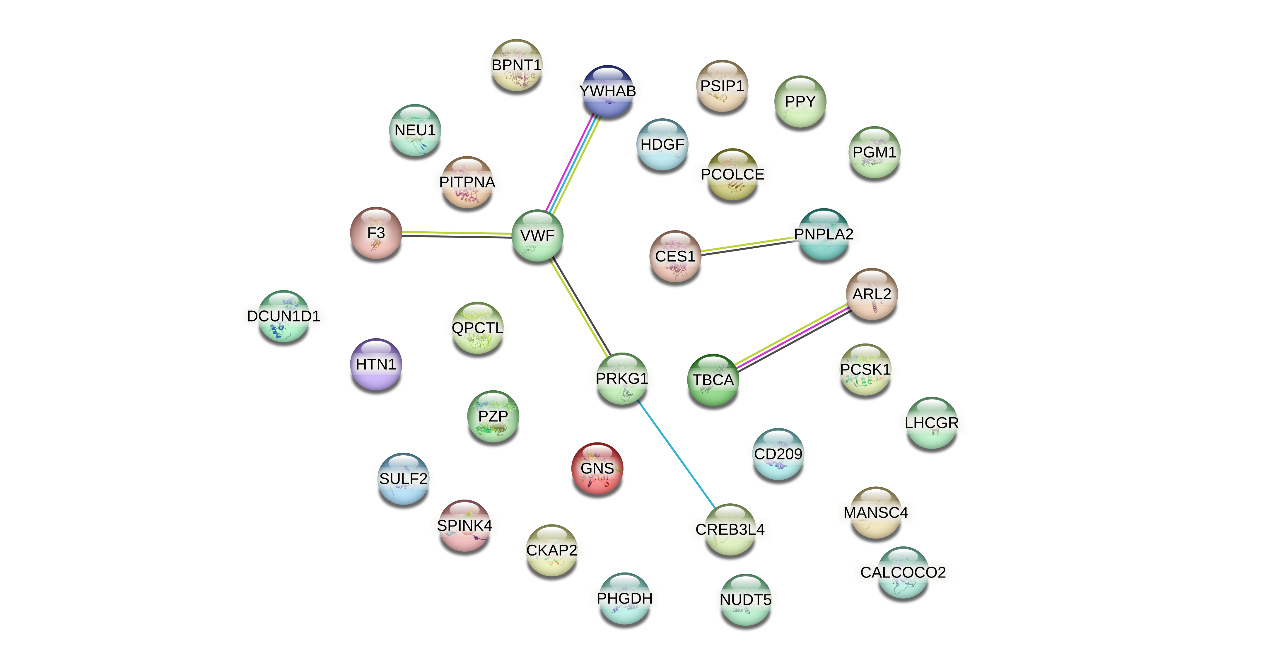


Supplementary Figure 2: Protein-protein interaction networks of the cis/trans Mendelian randomization-prioritized proteins.
